# Developing questionnaires for students’ evaluation of individual faculty’s teaching skills: A Saudi Arabian pilot study

**DOI:** 10.4103/1319-1683.71991

**Published:** 2010

**Authors:** Abdullah M. Al-Rubaish, Sheikh Idris Abdel Rahim, Ammar Hassan, Amein Al Ali, Fatma Mokabel, Mohammed Hegazy, Ladé Wosornu

**Affiliations:** *College of Medicine, University of Dammam, Dammam, Kingdom of Saudi Arabia*; 1 *Prince Mohamed Research Center, University of Dammam, Dammam, Kingdom of Saudi Arabia*; 2 *Colleges of Medicine & Nursing, University of Dammam, Dammam, Kingdom of Saudi Arabia*; 3 *Quality Management Unit, University of Dammam, Dammam, Kingdom of Saudi Arabia*

**Keywords:** Student evaluation of teaching effectiveness, student evulation of faculty teaching skills, academic accreditation, faculy personal portofolio, Saudi Arabia

## Abstract

**Background:**

The National Commission for Academic Accreditation and Assessment is responsible for the academic accreditation of universities in the Kingdom of Saudi Arabia (KSA). Requirements for this include evaluation of teaching effectiveness, evidence-based conclusions, and external benchmarks.

**Aims:**

To develop a questionnaire for students’ evaluation of the teaching skills of individual instructors and provide a tool for benchmarking.

**Setting:**

College of Nursing, University of Dammam [UoD], May-June 2009.

**Materials and Methods:**

The original questionnaire was “Monash Questionnaire Series on Teaching (MonQueST) - Clinical Nursing. The UoD modification retained four areas and seven responses, but reduced items from 26 to 20. Outcome measures were factor analysis and Cronbach’s alpha coefficient.

**Results:**

Seven Nursing courses were studied, viz.: Fundamentals, Medical, Surgical, Psychiatric and Mental Health, Obstetrics and Gynecology, Pediatrics, and Family and Community Health. Total number of students was 74; missing data ranged from 5 to 27%. The explained variance ranged from 66.9% to 78.7%. The observed Cornbach’s α coefficients ranged from 0.78 to 0.93, indicating an exceptionally high reliability. The students in the study were found to be fair and frank in their evaluation.

## INTRODUCTION

The accreditation body charged with academic accreditation of universities recently introduced in the Kingdom of Saudi Arabia is the National Commission for Academic Accreditation and Assessment (NCAAA). University of Dammam (UOD) was one of the first to be involved in the process.[1]

Of the 11 areas identified by NCAAA for evaluation according to internationally accepted standards of good practice, “Students’ Learning and Teaching” is considered of primary importance.[2] Requirements include: “A comprehensive system for evaluation of teaching effectiveness, including but not limited to student surveys.”[3]

The NCAAA “Course Evaluation Survey” (CES) evaluates the effectiveness of teaching in each course as a unit. However, there are other NCAAA requirements. First, “Faculty maintain portfolio of evidence of evaluation, and, of strategies for improvement.”^3^ Second, “analyses and conclusions should be based on valid evidence rather than subjective impressions.”[4] Third, benchmarks should include external comparison.[5]

Informative and important as they are, these directives are not sufficient for comprehensive evaluation of instructor’s individual professional areas of strength and weakness in general, and teaching skills in particular. The development of valid and reliable questionnaires for completion by students anonymously on each instructor separately is an indispensable tool for the provision of an authentic judgment on the teacher’s individual potential and aptitudes. This input for the evaluation of instructors’ teaching skills should preferably be focused each time on a single area of teaching skills.

Student Evaluation of Teaching Effectiveness (SETE) has been criticized on several grounds.^6^ Traditionally, it is regarded as sensitive. The controversy begins with questioning the validity of students’ evaluation of their professors’ teaching skills.[7–9] Teaching in universities is a complex and multi-dimensional task.[10] Another potential bias against SETE is that, it might induce leniency in the grades assigned to students among other factors.[11,12]

### Aim

The primary aim of this study was to develop a valid and reliable instrument for students’ evaluation of the teaching skills of individual instructors. A secondary aim was to provide a potential tool with which to benchmark teaching skills among different institutional settings. This paper reports initial results on the teaching skills of clinical nursing instructors.

## MATERIALS AND METHODS

### Study population

The study was carried out in the College of Nursing, UoD in the 2008/09 academic year. The focus of the study was students’ evaluation of each instructor’s teaching skills in clinical nursing courses. Students were assembled in their respective classes and the questionnaires were distributed to them. They were given sufficient time to respond to the questionnaire without prompting. Each group was supervised by an independent faculty member (i.e. one who was not being evaluated in that session.)

Throughout the study, care was taken to protect anonymity of evaluators i.e. the students, but not the evaluated i.e. the instructors.

### The questionnaire

The original questionnaire was the “Monash Questionnaire Series on Teaching (MonQueST) - Clinical Nursing.[13] It consists of four areas, 26 items and seven response options. These were: (1) All or almost all, (2) Most, (3) About half, (4) Only some and (5) Very few as well as (6) Entirely inappropriate and (7) Attended too few.

In the modification by UOD, the four areas and seven response options were retained, but the items were reduced from 26 to 20 [Table 1]. Response options 6 and 7 were put in a separate category because all students in the study were full-time, and their attendance at clinical instructions was mandatory. Accordingly, statistical analysis of the modified MonQueST was based on a 5-point scale relating to the first five response options.

Outcome Measures were factor analysis and Cronbach’s alpha coefficient.

**Table 1:** The monash questionnaire series on teaching (monquest)14 as modified by university of dammam

**The clinical teacher’s dealing with clients (N = 4)** 1.1 The teacher listened attentively to clients as they described their problems or concerns. 1.2 The teacher consulted clients to determine their nursing care needs and concerns. 1.3 The teacher sought clients’ reactions to the nursing care provided. 1.4 The teacher consulted other members of the health care team. **Clinical Teacher’s demonstrations and explanations (N = 4)[1]** 2.1 The teacher demonstrated nursing care procedures. 2.2 The teacher demonstrated and explained client assessment procedures. 2.3 The teacher used nursing situations I had seen to show how theory was related to clinical decisions. 2.4 The teacher reacted positively when students made comments or asked questions. **Students’ practice of nursing care (N = 4)[2]** 3.1 The teacher encouraged me to practice supportive and confident styles of communications with clients and members of the health care team. 3.2 It was possible for me to practice client assessment techniques. 3.3 I had chance to practice nursing procedures. 3.4 As my professional competence improved, the teacher’s supervision of my clinical activities gave me more responsibility. **The clinical teacher’s support and guidance of students (N = 8)[3]** 4.1 The teacher guided me on expected standards of personal and professional behavior. 4.2 The teacher gave me support and encouragement to practise nursing care procedures. 4.3 The teacher’s feedback showed how I might improve in nursing care procedures. 4.4 The teacher guided me on how to interact in awkward nursing care situations. 4.5 The teacher helped me identify aspects of my clinical work needing more practice. 4.6 The teacher directed me to references relevant to my practice. 4.7 It was possible to consult the teacher about any learning activity during the clerkship. 4.8 I had the teacher’s counseling if I had a stressful nursing situation with a client.
The Six Items Deleted from MonQueST.[1] Items 2.3 and 2.4;[2] Items 3.2, 3.4 and 3.7;[3] Item 4.7.
2.3 I understood the reasoning which the teacher used in interpreting client information.
2.4 The teacher’s explanation used concepts and principles which reflected my current level of learning.
3.2 The teacher encouraged me to practice supportive and confident styles of communications with members of the health care team.
3.4 When opportunities arose, it was possible for me to practice using client information to decide nursing care.
3.7 The responsibilities which were allocated to me extended my range of experiences.
4.7 I understood the arrangements should I have needed to consult with the teacher about any particular activity during the practice period.

### Statistical analysis

Data entry and analyses required SPSS version 13. Factor analysis was performed to measure the ability of the questions asked to relate in the actual construction that was intended for use. In this first step, the inter-item correlation was explored. This created a matrix of correlation of all items. Eignevalue and amount of variances explained was calculated for each item and for the different modules in the study.

At this stage, the risk of “singularity” had to be borne in mind (i.e. items that are perfectly correlated with R > 0.9). Therefore, two sub-types of items were identified: (a) Those that failed to correlate with others, and (b) Those which demonstrated singularity. This was a pre-requisite for the second step (i.e. reliability test) since the above items, if any, had to be excluded. A check for the normal distribution of the scores was also done.

Internal consistency reliability test (test-retest measure of reliability) was then performed by administering the same instrument to the same group of students for different instructors for each course. The internal reliability estimates were calculated using Cronbach’s alpha coefficient.[14] It provides a conservative estimate of reliability, and, generally represents the lower bound to the reliability of a scale item. Cronbach’s alpha coefficient greater than or equal to 0.70 was taken as an acceptable criterion for reliability of the scale.[15]

## RESULTS

At present, all the students and staff of the Nursing College are females. Seven courses from the Nursing Program were studied, namely: Fundamentals of Nursing, Medical Nursing, Surgical Nursing, Psychiatric and Mental Health Nursing, Obstetrics and Gynecologic Nursing, Pediatric Nursing, and Family and Community Health Nursing. There was one course from Level 2 and three each from Levels III and IV.

Response options 6 (“Entirely inappropriate”) and 7 (“Attended too few”) were dealt with as a separate category. The counted proportions were as follows: 0.20, 0.26, 0.30, 0.39, 0.49, 0.68 and 0.95% (Mean 0.65%). Thus, the selection of both options was numerically negligible.

Based on a 5-point scale, the total number of students was 74; missing data ranged from 5 to 27%.

### Factor analysis

All the 20 items of the employed questionnaire were entered in a factor analysis for each module, with a minimum of one eigenvalue for factor extraction and or 0.4 for item-to-factor loading. The procedure generated four areas in which all the 20 items were included. The explained variance ranged from 66.9% to 78.7%, depending on the module, except the “Fundamentals of Nursing”. In this module (sample size=74), inter-item correlations failed to emerge in 23% of paired items, and the explained variance was less than 54%. As a result, this module had to be excluded from further analysis.[16]

### Reliability

The internal consistency reliability was tested by Cornbach’s α coefficient for each of the four areas in each of the six modules with the individual student as the unit of analysis. The observed α coefficients ranged from 0.78 to 0.93, indicating an exceptionally high reliability. By convention, a lenient cut-off of 0.60 is common in exploratory research; alpha should be at least 0.70 or higher to retain an item in an “adequate” scale. Many researchers require a cut-off = 0.80 for a “good scale.”[15]

## DISCUSSION

All student evaluations are based on the hypothesis that students are the best experts to assess their teachers.[17,18] Nevertheless, Students Evaluation of Teaching Effectiveness (SETE) is controversial.[7–12,19–24] With the advent of NCAAA, institutions seeking academic accreditation in KSA will be required to apply SETE in the medium term. Writing from King Faisal University of Petroleum and Minerals in Dhahran, KSA, Siddiqi (2002) observed: “Proper questionnaire design has been cited as one of the key factors in the qualitative outcome of the exercise.”[18]

Questionnaires seeking students’ opinion should be reliable, valid and consistent, but also concise and adequate [Tables 2 and 3]. This is especially so if the area studied is traditionally regarded as sensitive such as students’ evaluation of their individual professors’ teaching skills. The exclusion of six items was informed by the logical and pragmatic approach. This demanded that all the key components in the original questionnaire be retained. Furthermore, the remaining 20 items which covered major aspects of teaching Clinical Nursing were more simply and clearly phrased for the students.

**Table 2:** A Summary of results from factor analysis on the modified monquest(6) questionnaire per six modules in the nursing program

Module	Sample size	Kaiser-Meyer-Olkin measure of sampling adequacy	Bartiett’s test of sphericity *P*-value	Eignevalue	% of amount of variance explained
Medical Nursing MDNU 317	58	0.810	< .001	14.6	72.10
Surgical Nursing MDNU	363 56	0.829	< 0.001	14.1	70.49
Psychiatric Nursing MDNU 365	65	0.831	< 0.001	15.74	78.7
OB/Gyne Nursing MDNU 422	65	0.902	< .001	13.38	66.9
Pediatric Nursing MDNU 454	54	0.774	< 0.001	14.05	70.4
FAMCO Nursing MDNU 461	70	0.877	< 0.001	14.41	69.5

**Table 3:** Crobanch reliability, items mean, standard deviation and ability to distinguish between classes for each scale of the modified monquest for six modules in the nursing program

Module/Scale	No of items	Unit of analysis	α Reliability	Mean	SD	ANOVA results (*P*-Value)
MDNU 317						
The clinical teacher’s dealing with client	4	Individual	0.854	16.03	3.5	0.022
Clinical teacher’s demonstrations and explanations	4	Individual	0.781	16.07	3	0.013
Students practice of nursing skills	4	Individual	0.823	15.97	3	0.303
The clinical teacher’s support and guidance of students	8	individual	0.905	31.17	6.8	< 0.001
MDNU 363						
The clinical teacher’s dealing with client	4	Individual	0.818	14.14	3.9	0.399
Clinical teacher’s demonstration and and explanations	4	Individual	0.828	15.02	4.1	< 0.001
Students practice of nursing skills	4	Individual	0.865	14.79	4.2	0.566
The clinical teacher’s support and guidance of students	8	Individual	0.932	29.05	8.5	0.009
MDNU 365						
The clinical teacher’s dealing with client	4	Individual	0.929	16.14	4.5	0.001
Clinical teacher’s demonstrations and explanations	4	Individual	0.922	16.85	4.2	0.107
Students practice of nursing skills	4	Individual	0.917	16.76	4	0.005
The clinical teacher’s support and guidance of students	8	Individual	0.956	32.85	8.4	0.003
MDNU422						
The clinical teacher’s dealing with client	4	Individual	0.805	14.98	3.3	0.008
Clinical teacher’s demonstrations and explanations	4	Individual	0.823	14.78	4	0.001
Students practice of nursing skills	4	Individual	0.809	14.53	3.7	0.319
The clinical teacher’s support and guidance of students	8	Individual	0.933	28.24	8.1	0102
MDNU 454						
The clinical teacher’s dealing with client	4	Individual	0.854	15.72	4.2	< 0.001
Clinical teacher’s demonstrations and explanations	4	Individual	0.853	16.98	3.5	< 0.001
Students practice of nursing skills	4	Individual	0.688	16.24	3.1	0.045
The clinical teacher’s support and guidance of students	8	Individual	0.934	32.73	7.3	< 0.001
MDNU 461						
The clinical teacher’s dealing with client	4	Individual	0.852	15.16	3.4	0.150
Clinical teacher’s demonstrations and explanations	4	Individual	0.832	15.55	3.5	0.001
Students practice of nursing skills	4	Individual	0.883	15.56	3.9	0.285
The clinical teacher’s support and guidance of students	8	Individual	0.922	31.32	7.1	0.274

Hence, it was gratifying to note that, the reduction of the items from 26 in the original instrument to 20 in the present version did not result in a significant reduction in reliability, validity or consistency of the instrument. It rendered the modified version more concise and suitable, for use in our local socio-cultural setting. It was therefore, fit for the intended purpose: that of readily providing valid, objective data.

Another issue for discussion is the minimum number of students required for an assessment of teaching to be valid.[25] In a recent publication, Chenot, Kochen and Himmel used a cut-off point of five students.[26] Thus, the number of students in this study was considered adequate, especially for a pilot study.

The modified MonQueST demonstrated another useful attribute: the ejection of one module as a result of statistical scrutiny: “Fundamentals of Nursing”. This outcome was subsequently validated by the Course Supervisor who pointed out that in actual delivery, the course was more theoretical than practical. This observation also confirmed that the students in the study were mature, fair and frank in their evaluation.

The final issue for discussion is the intended use of the results of such studies. Siddiqi raised a veiled objection: “It gets too much weight for contractual/job evaluation.”[18] Salsali concluded from an Iranian perspective that: “Systemic and continuous evaluation as well as staff development should be the primary goal.”[27]

It was clear from the beginning that results can be used for the three stated aims of the study. First, it was to help satisfy requirements of NCAAA that faculty maintain evidence of evaluation, and that analyses and conclusions were based on valid evidence.[3,4] Secondly, it could be used formatively. This includes needs assessment for the teaching skills component of professional development of individual faculty. Thirdly, it could form a link for external Institutional benchmarking.[5]

The University of Dammam is in a transitional phase of academic accreditation. This demands that we refine and customize various tools including questionnaires. These results remain to be confirmed. It is hoped that field-testing will widen its application by refining them for use in other colleges of University of Dammam, the Eastern Province as well as KSA and Gulf States.

## CONCLUSIONS

The qualitative aspects of the study have not been determined. In other words, the students’ opinion as well as the peers of those evaluated have to be authenticated by the Dean of College. This will be the subject of separate study. Pending authentication, two tentative conclusions can be drawn. The modified MonQueST for Clinical Nursing has been found to be efficient, adequate, reliable and consistent. It can be used formatively as stated above. However, it remains subject to ongoing review and optimization, and may only be used as part of the range of faculty evaluation tools as required by NCAAA.
